# Passive Radar for Opportunistic Monitoring in E-Health Applications

**DOI:** 10.1109/JTEHM.2018.2791609

**Published:** 2018-01-25

**Authors:** Wenda Li, Bo Tan, Robert Piechocki

**Affiliations:** CSN laboratory, School of Computer Science, Electrical and Electronic Engineering, and Engineering MathematicsUniversity of BristolBristolBS8 1UBU.K.; School of Computing, Electronics, and MathsCoventry UniversityCoventryCV1 5FBU.K.

**Keywords:** Passive sensing, opportunistic sensing, doppler radar, e-health, breathing detection, activity recognition

## Abstract

This paper proposes a passive Doppler radar as a non-contact sensing method to capture human body movements, recognize respiration, and physical activities in e-Health applications. The system uses existing in-home wireless signal as the source to interpret human activity. This paper shows that passive radar is a novel solution for multiple healthcare applications which complements traditional smart home sensor systems. An innovative two-stage signal processing framework is outlined to enable the multi-purpose monitoring function. The first stage is to obtain premier Doppler information by using the high speed passive radar signal processing. The second stage is the functional signal processing including micro Doppler extraction for breathing detection and support vector machine classifier for physical activity recognition. The experimental results show that the proposed system provides adequate performance for both purposes, and prove that non-contact passive Doppler radar is a complementary technology to meet the challenges of future healthcare applications.

## Graphical abstract

This paper proposes passive Doppler radar as a non-contact sensing method to capture human body movements and to recognize respiration and physical activities in e-Health applications. The system uses existing in-home wireless signals as the source to interpret human activity and an innovative two-stage signal processing framework. Experimental results show that passive radar is a novel solution for multiple healthcare applications.

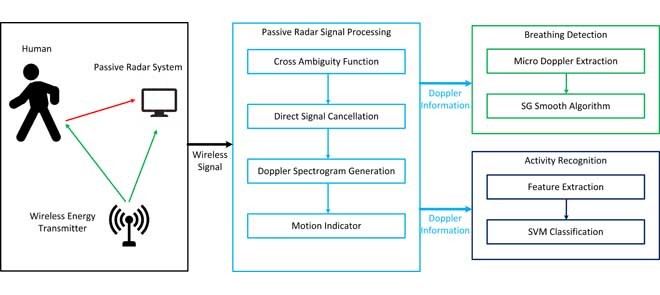

## I. Introduction

Aging and chronic diseases become prominent societal challenges and pose a significant strain on public finances and life quality. The resolving of these challenges can be shifted to understanding the life patterns of individuals. For example, the vital signs can be used for monitoring medical problems [1] and the activity recognition can be used to forecast and prevent many chronic diseases [2], [3]. However, capturing such information is a challenging task. Many diverse approaches have been proposed from both industry and academia for the in-home healthcare informatics, and can be generally divided into wearable sensor [4], [5] and non-contact sensors [6]–[9]. Currently, wearable sensors are considered as the primary technique as a source of rich information pertaining to health. However, wearable sensors are not suitable for everyone. Some patient groups like people with skin diseases, Parkinsons and infants are discouraged from wearing such sensors [6], [10]. Thus, the non-contact sensors may be the only solution for those who cannot wear any device on body.

The efficacy of non-contact in-home monitoring systems have been successfully demonstrated by various technologies. Pyroelectric Infra-Red Detector (PIRD) for the elderly in [11]. Camera-based action analysis system developed in [7], [12] gives good activity classification result, but difficult to be deployed in bedrooms and bathrooms for privacy concerns. Alternatively, human movement recognition by using ultrasound echo had been developed in [8] but suffers poor range coverage. It requires a large number of sensors to cover a single house. Both Continues Wave (CW) radar [13] and narrowband Doppler radar [14] have been also used for human gait analysis and fall detection with adequate performance. Works in [15] and [16] propose the Ultra-Wide-Band (UWB) radar system and show advantages in coverage, accurate localization and vital signs monitoring like breathing detection and the ability to identify breathing patterns. However above active radar techniques are facing several limitations like spectrum licensing and infrastructure deploying problem. In this paper, we propose a passive wireless sensing system for in-home healthcare which relaxes the aforementioned constraints.

Given all above constraints, we raise two questions: Is it possible that a non-contact and ’passive’ detection system can be accurate enough for activity capturing and breathing detection for healthcare applications? Furthermore, how to design the system that can accommodate multiple motion levels wireless detection function? To answer those questions, we first investigate the existing in-home RF signals and the patterns induced by human movements can be potentially used for non-contact human sensing. Secondly, regarding the meaning of “multiple motion levels”, we consider two situations for the user: static or moving status. We define breathing detection as static status sensing and activity recognition for moving status sensing. To deliver the idea, we face two major challenges: 1) How to design the system that uses available in-home wireless signal as the source for sensing the user in a typical residential RF environment? 2) How to merge the breathing detection and activity recognition into one system without obscuring the body motion with breathing induced chest motion?

To address these challenges, a passive Doppler radar system is proposed to capture the human body motion. The system leverages the Doppler information contained in the reflected signal from the human body to glean information related to human movement. To discriminate between ***micro*** chest motion (for breathing detection) and ***macro*** body movements (for activity recognition), we design the system with adaptive Doppler resolution to cope different monitoring scenarios: 1) if the person is in static status, the passive system starts the “breathing” mode with high Doppler resolution; 2) otherwise, the activity recognition mode is enabled with low Doppler resolution.

In this paper, we design and implement a passive radar system for human sensing. Our work in [17] demonstrates the capability of using narrow band energy harvesting signal for respiration detection. The work in [18] shows the activity recognition and activity level indication capability of WiFi data signal and WiFi beacon signal. The work in [19] explores the unsupervised learning approach for activity recognition. However, above systems [17]–[19] are all designed for a specific purpose. While in this paper, we proposed a cognitive mechanism to allow the system can work adaptively for different purposes in the residential house. In radar community, this adaptive sensing is often called cognitive sensing. For breathing detection, we adopt the micro Doppler extraction [17] to extract the breathing signal with more quantitative measurements from three volunteers. For activity recognition, we explore the full potential of measured Doppler information to precisely classify the activity with following procedure: (1) extract the features vectors from Doppler spectrogram; (2) study the difference between the activities with training sets; (3) classify the testing sets with pre-built models. Experiment results demonstrate that passive system is capable of both accurate breathing detection at a realistic distance and achieving a highly accurate activity recognition. Comparing with the state of the art [2], [6], [9], [20] and our previous work [17]–[19], the proposed system demonstrates the following:

• We design and implement a novel passive radar sensing system which is capable for both breathing detection and activity recognition. Comparing with our previous works [17]–[19], it is demonstrated to be more robust for real-world application due to the adaptive multiple motion levels design.
• We investigate the micro Doppler feature from chest movement and propose a fine gained micro Doppler extraction algorithm for tiny chest movement detection. Simultaneously, our proposed system is more tolerance in user’s posture varying and offers wider detection range when comparing with [21], [22].
• Different from previous UWB based approach, we investigate the feasibility of activity recognition driven by passive radar technique in this paper. The results show more than 85% classification accuracy can be achieved for six typical in-home activities, which are adequate for healthcare purpose.

This paper starts with the description of system design and the experiment design in Section II. Section III outlines the time-frequency passive radar signal processing. The fine-grained Doppler extraction algorithm and experiment result of breathing detection are outlined in section IV. Section V shows the classification method and experiment result for activity recognition. Conclusions will be given in section VI.

## II. System Scope and Experiment Layout

**FIGURE 1.**
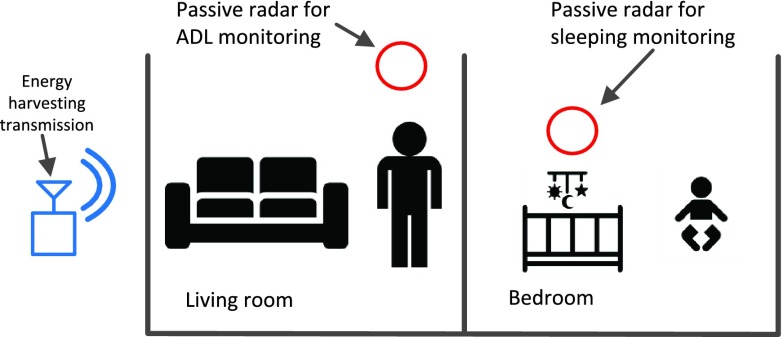
Potential deployment of the proposed passive radar system in e-Health application.

To understand the role of passive radar in healthcare systems, let us consider the house shown in Fig. 1, where a RF transmitter is deployed. This is common for current and future house design, for example, a WiFi Access Point (AP) for communication or wireless energy transmitter for energy delivery. Meantime, an adult is staying in the living room and a baby is sleeping in the bedroom. Regarding in healthcare purpose, the Activities of Daily Living (ADL) information is essential for the adult as it provides a valuable reference about his health condition which is highly correlated with his physical activities [23]. This can be achieved by classifying each activity via analyzing the difference in Doppler spectrogram. For the baby, overnight breathing monitoring is important as it helps to identify the sleeping patterns which can be used to prevent sleep-related diseases such as Sudden infant death syndrome (SIDS) and Obstructive Sleep Apnea (OSA) [24]. The chest expansion (inhale motion) and shrinkage (exhale motion) will output a positive and negative Doppler shift from our passive radar system.

**FIGURE 2.**
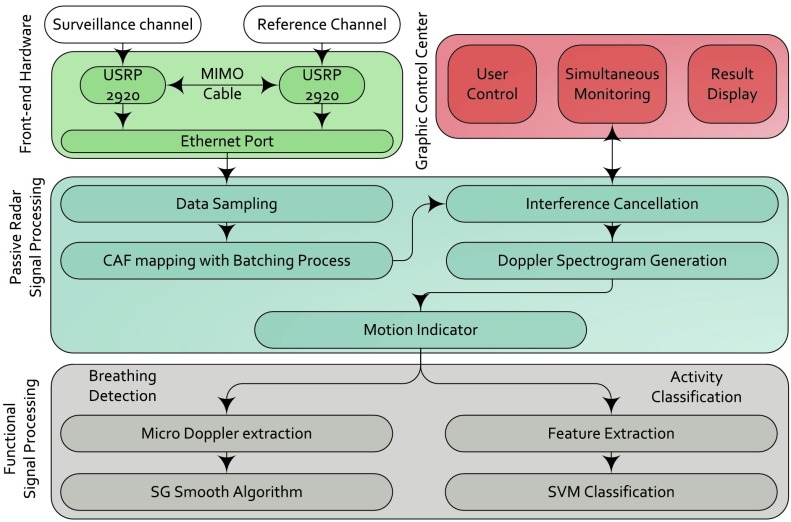
Block diagram for system overview.

The key feature of this system is the capability of detecting both breathing and activities on one platform. The main challenge for this task is the adaptable detecting sensitivity from few centimeters (chest movement) to meters (body motion). Previous work addresses this challenge by using various sensors, for example, [3] uses multi-sensor system (including pressure, sound, contact) to access the adult’s ADL information and wearable sensor for vital signal detection. In contrast, our system only uses passive radar based sensor to achieve multiple level activities detection by adjusting Doppler resolution. This presents a significant innovation in the design of passive radar for e-Healthcare applications.

**FIGURE 3.**
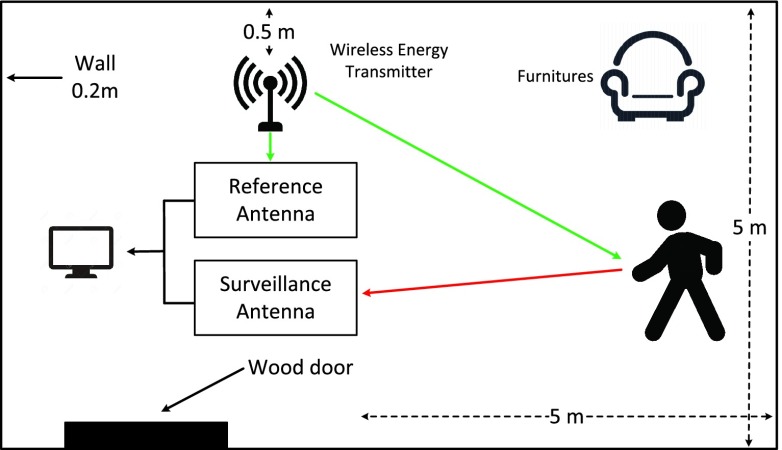
Experiment layout.

The proposed system is based on our previous work in [17]. The front-end hardware contains two tunable RF transceivers NI USRP-2920 [25] for acquiring the signal from reference and surveillance channels respectively. Reference channel usually contains the signal from source while the surveillance signal mainly contains the reflections from moving human. A MIMO-cable was used to share the clock signal between two USRPs for synchronizing the reference and surveillance signals. The signal processing is designed into two parts: passive radar processing and functional signal processing. The passive radar signal processing includes signal sampling, Cross Ambiguity Function (CAF) mapping, interference cancellation and Doppler spectrogram generation [26]. Then a motion indicator has been designed to classify the static and moving period base on the power from Doppler spectrogram. Breathing detection and activity recognition are designed as two parallel functions of the proposed system. The overall system architecture is shown in Fig. 3 in a high clutter environment with lots of furnitures and equipments around.

The experiment was carried out in the lab in University of Bristol. Three volunteers (males, age 38, 26 and 27) have been involved. The experiment layout is shown in Fig. 3. A wireless energy transmitter [27] was used as the illuminator which operates at 915 MHz ISM band. It provides a more powerful (up to 30 dBm) and continues wireless signal compared to the WiFi, which mostly depends on the network usage [18]. Wireless energy will be widely used for battery-free sensors in future residential context [27]. A surveillance antenna was pointed to the volunteers to capture the reflected signal, and a reference antenna was pointed to the wireless energy transmitter to recreate transmitted signal. Both are PCB antennas with size of 164 mm $\times \,\, 20$ mm $\times \,\, 1$ mm and 1.5 dB gain. During the experiment, volunteers were asked to perform both static period and moving period within an area of 5 $\times $ 5 meters including 117 (13 situations $\times \,\, 3$ repetitions $\times \,\, 3$ volunteers) measurements from breathing detection and 180 (6 activities $\times \,\, 10$ repetitions $\times \,\, 3$ volunteers) measurements from activity recognition. The details of those measurements will be explained later in Section III and IV respectively.

## III. Passive Radar Signal Processing

Starting with the signal source (wireless energy transmitter) used in our experiment, it transmits a direct sequence spread spectrum (DSSS) signal in sub 1GHz ISM band. The DSSS signal can be generally expressed as (1):

\begin{equation*} x(t) = e^{j2\pi f_{c} t} \sum _{i=1}^{N} e^{j\varphi _{i}}p(t) \end{equation*}

where $N$ is the number of data bits, $f_{c}$ is the carrier frequency, $p(t)$ is the pulse shaping function and $x(t)$ is the transmitted signal. The received signal consist of both direct signal, target reflections and reflections from other stationary paths. The echo from a cluster or a moving person can be described by a delayed and Doppler shifted transmitted signal. The received signal $y(t)$ can be presented as sum of signals from all propagation paths (2):

\begin{equation*} y(t) = \sum _{l}^{} A_{p} e^{j2\pi f_{c} f_{d} t} x(t-\tau) + w(t) \end{equation*}

where $l$ is the number of arrival paths, $A_{p}$ is the attenuation of each path, $\tau $ is the time delay and $f_{d}$ is the Doppler shift due to the moving target. $w(t)$ is the additive white Gaussian noise (AWGN). In a passive radar system, the transmitted signal $x(t)$ is received by the reference channel (between the reference antenna and signal source), whereas the reflected signal $y(t)$ is received by the surveillance channel (between the surveillance antenna and surveillance area). By correlating the signal from reference and surveillance channels, the range and Doppler information can be derived by using CAF mapping. To reduce the computational complexity, we also apply the batching process [28] to this calculation:

\begin{equation*} CAF (\tau, f_{d}) = \sum _{k=0}^{N_{b}-1} \int _{0}^{T} x_{i}(t) {y^{*}_{i}{(t-kT_{B}-\tau)}} e^{j2\pi f_{d} f_{c} t} dt\quad \end{equation*}

where $n$ is the index of batch, $T$ is the integration time, $n_{b}$ is the number of batch and limited by max detectable velocity $V_{max}$, $T_{B}$ is the length of each batch and normally is chosen as $T_{B}=T/n_{b}$ and $\left \lfloor{ * }\right \rfloor $ means the complex conjugation. The range resolution of passive radar is defined as $\Delta R=c/2B$, however in the case of narrow-band wireless energy harvesting signal (20 MHz), the range resolution is limited at 7.5 meters which is too coarse for indoor scenario. On the other hand, Doppler resolution is inversely proportional to the integration time as $\Delta f=1/T$ which allows us possible to adjust its resolution.

The CAF mapping between the reference and surveillance channels causes unwanted peak in the zero-Doppler bin due to the strong direct signal from the source and reflected signal from surrounding stationary objects. To reveal weaker dynamic echoes from these interferences, the CLEAN algorithm [29] has been used by subtracting the scaled self-ambiguity surface $CAF_{self} (\tau,f_{d})$ of the reference signal from original CAF surface. The cleaned CAF mapping $CAF(\hat {\tau },\hat {f_{d}})$ can be then obtained by (4):

\begin{equation*} CAF(\hat {\tau },\hat {f_{d}}) = CAF(\tau,f_{d})-\alpha ^{k} CAF_{self}(\tau,f_{d}) \end{equation*}

where scaling factor $\alpha ^{k}$ is the maximum absolute value of $CAF(\tau,f_{d})$ amongst zero Doppler line. Doppler spectrogram is normally used to analyzed the activity, by picking column containing the maximum absolute value in the CAF mapping, the spectrogram can be generated as:

\begin{equation*} D(f_{d},n)= \sum _{n=0}^{k-1} arg_{f_{d}} \left \{{ max CAF_{n} (\hat {\tau },\hat {f_{d}}) }\right \} \end{equation*}

where $CAF_{n}$ represents one single CAF mapping, $k$ is the total number of recorded CAF mapping in a spectrogram, $arg_{f_{d}} {.}$ donates the column with maximum peak. In this system, both breathing detection and activity classification are based on this Doppler spectrogram and will be presented in Section VI and V.

**FIGURE 4.**
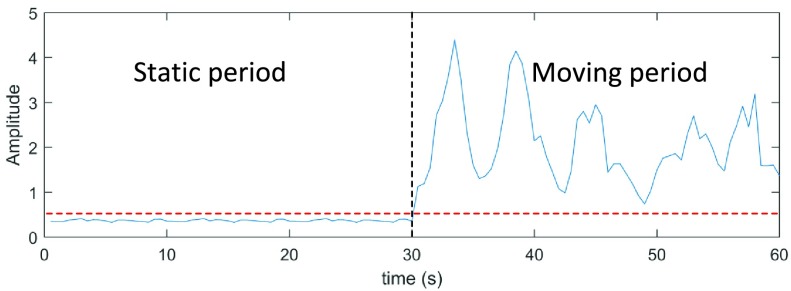
An example of Doppler power for static and moving period.

Afterwards, a motion detector which aims at automatically switching between static and moving period has been designed. This is achieved by calculating the Doppler power from spectrogram, which is treated as a pulse detection. A 30-seconds example of Doppler power obtained from both static (a person standing still at 40 cm away from antenna) and moving period (a person randomly walking in the surveillance area) are shown in Fig. 4. As can be seen, there is an obvious difference in the amplitude between the static period and moving period. This is because of the Doppler power from a static person is very low compared to a moving person. Based on this phenomenon, we set the reference level at 10% of the waveform amplitude (the red line) to discriminate the static and moving period. Overall 98.65% accuracy rate is observed from 297 measurements (117 from static period, and 180 from moving period). Note that, since we use the Doppler power instead of RSS so that the static background will not affect the performance of motion indicator.

## IV. Breathing Detection

This section describes the algorithm and experiment results for breathing detection. First, we demonstrate the concept of obtaining breathing signal from Doppler information. Then three experiments including accuracy versus distance, accuracy versus orientation and accuracy under through-wall scenario are carried out to verify the feasibility of proposed system. A chest belt (equipped with a gas pressure sensor [30]) is used to provide the ground truth for breathing rate.

### A. Signal Processing for Breathing Detection

The main challenge of breathing detection is extracting micro Doppler shift from reflections produced by unobvious chest movement. Therefore, high Doppler resolution is required which can be achieved by extending the integration time. However, the Doppler shift has to be assumed constant during the period of integration time in this hypothesis. In this case, increasing of integration time will blend both positive (inhalation) and negative (exhalation) Doppler shifts in one CAF range bin and mislead further analysis. Thus we use the micro Doppler extraction method proposed in our previous work [17] to extract the breathing signal from the Doppler spectrogram as (6):

\begin{equation*} \Psi (n) = \sum _{n=0}^{k-1}\sum _{i=0}^{j-1} f^{i}_{mD} (n) * \left \lceil{ \frac {j}{2} - i }\right \rceil \end{equation*}

where $\Psi (n)$ is an introduced parameter to represents the intensity of shape difference, $i$ is the size of one column in $D(f_{d},n)$ and $i$ is the index of the column. When the target is in stationary, $f_{m}D^{i}(n)$ can be obtained directly from $D(f_{D},n)$ as it only contains chest movement.

The SG smoothing filter [31] has been applied after micro Doppler extraction for purpose of noise removing. According to [31], a least square polynomial $p(n)$ length of N is applied to the signal with a moving window of size $2M+1$, the equation can be stated as:

\begin{equation*} p (n) = \sum _{k=0}^{N} a_{k} n^{k} \end{equation*}

where $a_{k}$ is the $\boldsymbol {k}$-th coefficient of the $p(n)$. The output value is smoothed at the central point of $n=0$. This procedure is repeated over the stream of micro Doppler $\hat {\Psi (n)}$, the denoised breathing signal can then be extract as:

\begin{equation*} \hat {\Psi (n)} = \sum _{m=n-M}^{M} h \left [{ n-m }\right] \Psi (m) \end{equation*}

Typically the normal breathing rate ranges from 12–20 breaths per minute [1], in our system, the size of window length of SG filter is set at two seconds on the micro Doppler to smoothing the noisy data while conserving the shape and weight of the peak. The efficiency of the micro Doppler extraction and smooth filter will be included in next section.

### B. Detecting the Breathing Signal

**FIGURE 5.**
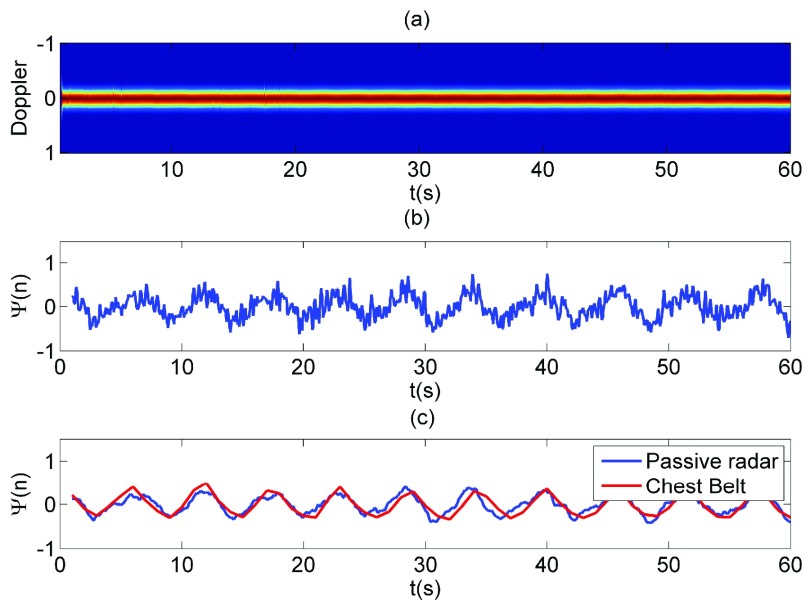
The Effective of Micro Doppler Extraction on Breathing Detection a) spectrogram of breathing motion, b) micro Doppler without SG filter, c) micro Doppler with SG filter and ground truth: chest belt signal.

We provide a 60 seconds measurement to demonstrate the process breathing signal. The first step is to obtain a Doppler spectrogram (equation 5) as shown in Fig. 5(a). As can be seen, the Doppler trace remains at zero Doppler bin due to the insufficient of Doppler resolution which can hardly be used for breathing detection. Then the micro Doppler extraction (equation 6) has been applied to the Doppler spectrogram, the result is shown in Fig. 5(b). The breathing signal is then revealed from the Doppler spectrogram but presents a noisy waveform. Then a SG smooth algorithm (equation 8) is applied to smooth the breathing signal. The output breathing signal (blue line) is shown in Fig. 5(c) and have a high correlation comparing with the ground truth from chest belt (red line).

### C. BREA Detection Accuracy

Three different experiments have been conducted to present the measuring accuracy. Each experiment was repeated for three times by each volunteer and last for 30 seconds. We output the breathing signal from the passive system with breathing curve measured from chest belt simultaneously and normalize the chest belt signal to the micro Doppler signal. The correlation-coefficient (R) and mean-square-error (MSE) between the micro Doppler signal and chest belt signal are calculated as performance measurement. In addition, we reduce the sampling rate from passive radar to match the chest belt sampling rate in order to calculate the R and MSE.

#### 1) Accuracy Versus Distance

**FIGURE 6.**
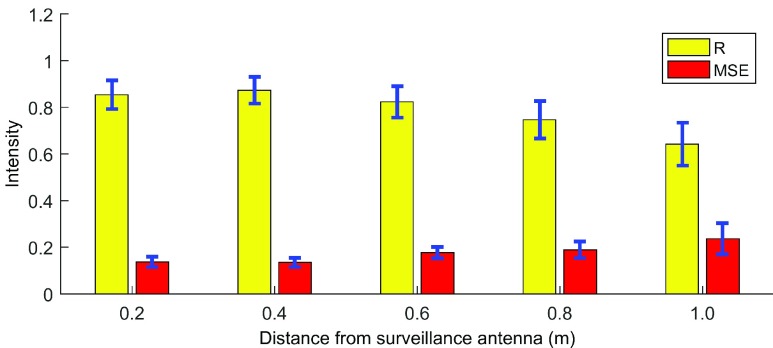
Breathing accuracy versus distance between target and surveillance antenna.

In this experiment, we investigate the ability of the passive system to monitor the breathing rate at different distances from the surveillance antenna. The subject sits on a chair at marked locations which are located 0.2m to 1.0m away from the antenna. The averaged R and MSE are shown in Fig. 6 with variability in different measurements (blue line). As expected, the MSE increases and R decreases with the distance increases. This drop in accuracy is due to the decreasing in signal-to-clutter-ratio (SCR) as the result of increased distance between chest and surveillance antenna. In addition, we further test the breathing rate by applying Fast Fourier transform (FFT) to the breathing signal. The results show the system is able to correctly detect breathing rate with average 87% (39/45) with up to 1m distance.

#### 2) Accuracy Versus Orientation

**FIGURE 7.**
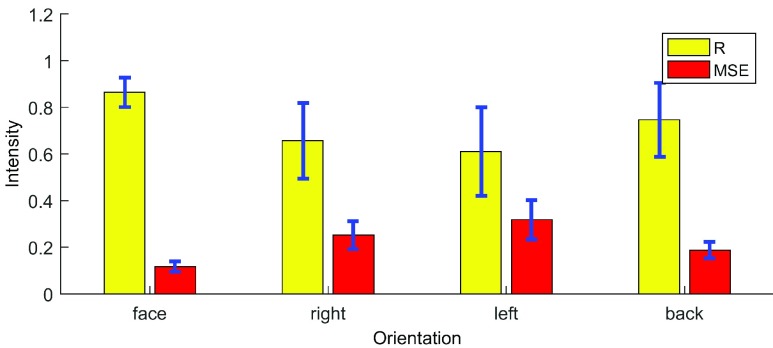
Breathing accuracy versus four orientations between target and surveillance antenna at 40 cm distance.

In the second experiment, we would like to test the performance when the volunteer does not face the surveillance antenna. For this purpose, volunteers were asked to stay 40 cm away from the antenna with four different orientations including: i) facing to the antenna, ii) right perpendicular to the antenna, iii) left perpendicular to the antenna and iv) back to the antenna. We plot the R and MSE for all four different orientations in Fig. 7. As it can be seen, when the volunteer faces the surveillance antenna, the R is highest with more than 0.8 and drops to around 0.6 at left orientation. The left orientation has also the highest MSE at 0.3. This shows the detection performance is better when the subjects face or back orientations. The reason is that although the chest motion also effects in the perpendicular direction, it has smaller expansion compare to the front and back. The average accuracy of this experiment is 86% (31/36).

#### 3) Accuracy with Separated Signal Source

One of the disadvantages of passive radar is its complex geometry that restricts the detection performance [26]. For the separated signal source case, the geometric problem becomes further complicated as the location of surveillance antenna, reference antenna, target and Signal Source can be either in the same room or different room [32]. Although we can place the surveillance antenna and reference antenna in a separate room. However, from the application aspect, this setup could largely increase the system cost. Therefore, we consider our passive system as one single unit. Then the geometry problem is reduced to three components: target, passive radar system and signal source. Considering the operation scenario in Fig. 1, the user and monitoring system are more likely to be within one room whereas the wireless energy transmitter is in another room.

**FIGURE 8.**
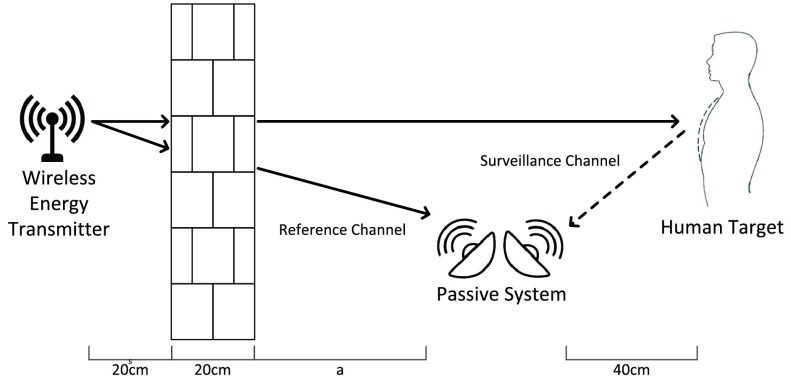
Experiment layout of passive radar sensor for separated signal source breathing detection.

In this experiment, we examine the accuracy of breathing detection versus distance between the passive system and wireless energy transmitter under a separated scenario. The experiment layout is shown in Fig. 8. We place the wireless energy transmitter to another room and place it 20cm to the brick wall (about 20cm width). Volunteers were asked to stay approximate 40cm to the surveillance antenna in all four measurements in this experiment.

**FIGURE 9.**
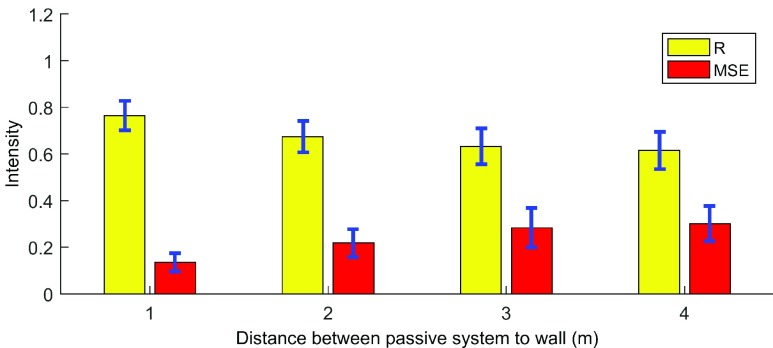
Breathing accuracy under separated signal source scenario versus distance between passive system to the wall.

We test the performance of the passive system to the brick wall ranges from 2m to 5m with 1m interval. The measured results are shown in Fig. 9. As can be seen, the R drops as increase in distance, but maintain more than 0.65 until 4m. And the MSE increases from 0.18 at 1m until 0.35 at 4m. The breathing rate accuracy among all measurements in this experiment is obtained at 90% (33/36). The reason for this phenomenon is that the distance between the target and surveillance antenna is constant so that the detected breathing signal could maintain at an acceptable level. This result shows the proposed system can cover a large area and has the potential for a whole house monitoring.

From the experiment results, we can see that a passive radar system can be easily deployed around the bed to monitor the breathing of personnel and obtain the sleeping pattern for further research.

## V. Activity Recognition

**FIGURE 10.**
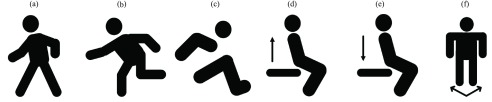
Illustration of six different human activities to be recognized by the system.

Another function of our system is the activity recognition which is designed for the situation when target is moving. To demonstrate this capability, total 6 different activities have been selected according to [2] that provide valuable health information: *(a) walking, (b) running, (c) jumping, (d) standing, (e) sitting and (f) turning* as present in Fig. 10. Those activities are basic human motions that valuable for various healthcare application [2]. In this experiment, each volunteer performs 10 datasets for each activity (total: $10\times 3\times 6=180$).

**FIGURE 11.**
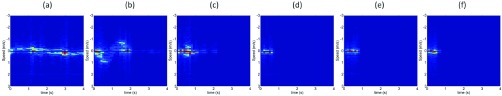
Demonstration of Doppler spectrogram for six activities.

Similarly to breathing detection, we use the Doppler information for activity recognition. The difference is body motions cause much larger Doppler shift comparing with chest movement. Therefore, the system will perform the activity recognition instead of the micro Doppler extraction in this function. Thus, we reduce the integration time $T_{i}$ to 1 seconds so that the Doppler information could briefly present the difference between each activity. Otherwise, the large integration time could result in noncontinuous or incomplete Doppler spectrogram. We collect the Doppler spectrogram from six activities with equation 5 and plot in Fig. 11. As can be seen, the Doppler spectrogram of (a), (b), (c) are visually different from that of (d), (e), (f) due to those activities are relative drastically and longer period. Although (d), (e), (f) only spans less than 1 second, there are still differences amongst their Doppler spectrogram. Previous work [9], [21], [33] show that a particular activity corresponds to a certain Doppler signature can be used for classification purpose by detecting pattern differences.

### A. Feature Selections

Since the Doppler spectrogram contains a significant amount of data, therefore it is necessary to reduce the dimension of data by extracting the feature component. The basic idea of feature extraction is to generate a feature vector $\boldsymbol {S}$ as the representation of the Doppler spectrogram $\boldsymbol {D}$ (generate from Equation 5), where $\boldsymbol {S}$ normally has lower dimension but with most significant features. In this work, we select three popular features, Singular Value Decomposition (SVD), Principle Component Analysis (PCA) and Physical Features (PF), that have been widely used in radar-related activity recognition applications. Those features are generated from the same dataset with a time window of 4 seconds.

#### 1) Singular Value Decomposition

SVD is a simple but powerful algorithm aims to reduce the data dimension. The SVD decomposition is given by:

\begin{equation*} \boldsymbol {D} = USV^{T} \end{equation*}

where $S$ is the diagonal matrix with singular values of the Doppler spectrogram $\boldsymbol {D}$, $U$ is the left singular vector and $V$ is the right singular vector. $T$ represents the transpose operation. In this work, we extract the non-zero singular values from the diagonal matrix $S$. This indicates the reduced subsets $U_{r}$ and $V_{r}$ which are then used to calculate the SVD feature vector as $S_{svd}=U_{r} S_{r} V_{r}^{T}$.

#### 2) Principle Component Analysis

PCA is a popular feature extraction methods in radar area. Lets define the Doppler spectrogram after removed mean from each column as $\hat {\boldsymbol {D}}$. Then the covariance of $\boldsymbol {D}. \hat {\boldsymbol {D}}$ and corresponding eigenvectors $W$ are calculated. Afterwards the reduced PCA feature vector $\boldsymbol {S_{pca}}$ can be defined as:

\begin{equation*} \boldsymbol {S_{pca}} = \hat {\boldsymbol {D}} W_{L} \end{equation*}

where $W_{L}$ is the truncated version of $W$. The value of $L$ is defined base on the energy of eigenvalues of the matrix $\boldsymbol {D}. \hat {\boldsymbol {D}}$. In this paper, we select 90% of the total energy in PCA coefficients (first 10 eigenvalues).

#### 3) Physical Features

Another type of feature [19] contains the physical meaning from the Doppler spectrogram, for example, the duration of activity or bandwidth of Doppler peak. In this work, refers to our previous work [19], the physical feature vector $\boldsymbol {S_{ph}}$ includes six features as defined in Table 1.

**TABLE 1:** Physical Features

Feature	Description
$S_{ph}^{1}$	time duration
$S_{ph}^{2}$	maximum upper Doppler shift
$S_{ph}^{3}$	maximum lower Doppler shift
$S_{ph}^{4}$	peak-to-peak bandwidth
$S_{ph}^{5}$	mean power of Doppler
$S_{ph}^{6}$	standard deviation of Doppler power

Above feature vectors will be later used for classification purpose.

### B. SVM Classifier

After determining the features, a proper classifier is needed to classify those feature vectors into corresponding activity group. In this work, the support vector machine (SVM) is used because of the small number of available training set (total 180), whereas other methods like Bayesian or neural networks usually need a large amount of training data set. There are two major extensions of SVM for N-multiclass problem. The first solution was to convert N-class problem into a group of two-class problems and known as one-against-all [33]. The simplest method for this extension is to build N binary classifiers to distinguish one class from all the others. The winner is the class with the most significant value in from the classifier. The second solution is called one-against-one as presented in [34]. In this case, total $N*(N-1)/2$ classifiers are required to differentiate classes $P_{i}$ and $P_{j}$ where, $0<iN$ and $0<ji$ [2]. For each classifier, it assigns the vote to one of the two classes; the winner is the class with the most votes. The work [35] shows that one-against-one classifier outputs better performance than one-against-all classifier especially for a small number of sample. Although more classifying processing is needed, yet the smaller size of each linear problem in kernel that results in faster process. Thus in this work, the one-against-one SVM classifier has been used, which is built on the basis of LIBSVM [36]. With the kernel and penalty parameter, LIBSVM calculates the classification error for testing data based on the boundary from the training data. Every single classifier solves the unconstrained optimization problem as following:

\begin{equation*} \frac {1}{2}w^{T}w + C\sum _{i=1}^{n}\xi (w_{i}; S_{i}, y_{i}) \end{equation*}

where $w=$ is the optimal weight vector, $S_{i}$ is the i-th feature vector and $y_{i}$ is the class label. $C$ is the penalty parameter for misclassification, and $\xi $ is the convex loss function that allows to be employed in solving optimal weight vector, if $\xi = 0$ means the dataset are classified correctly without any error.

As we have 6 different activities, total 15 (_6_c_2_) SVM classifiers have been built.

### C. Classification Results

The recorded data is then separated into training set and testing set for SVM classifier. The number of data set was set with 20 for training set and 10 for testing set. To reduce the classification uncertainty of the recorded data, we perform 10 tests with random selection on the training and testing set.

**FIGURE 12.**
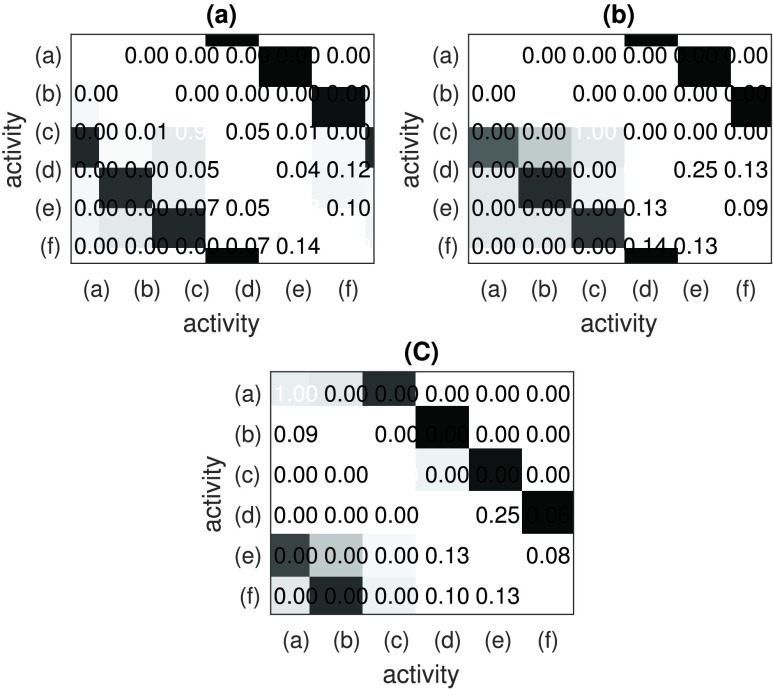
Confusion matrix for (a) SVD, (b) PCA and (c) physical features.

The obtained confusion matrices are given in Fig. 12 (a) SVD feature, (b) PCA feature and (c) physical features. As can be seen, the activity (a) has the best performance with 100% classification rate in all feature vectors. The second best is activity (c) with 100% in both PCA and physical features and 93% in SVD. Followed by activity (b), 100% in both SVD and PCA, and 91% in physical features. In comparison, activities (d), (e), (f) have downgrade performance and also contains lots of confused dataset into other classes. The best performance is shown in SVD with an average accuracy of 79% for three activities. The reason for this difference in recognition performance is because activities (d), (e), (f) have similar Doppler pattern and time duration which increase the difficulty in recognition. While activities (a) and (b) last longer in duration and activity (c) contains more expanded Doppler shifts.

**FIGURE 13.**
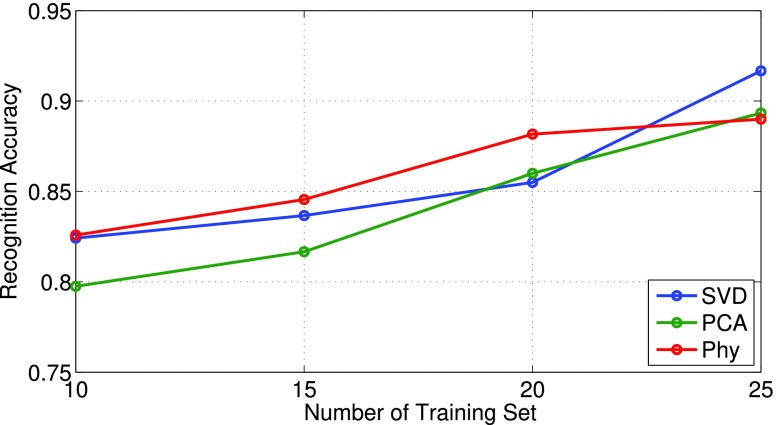
Classification accuracy versus different number of training set.

We further test the classification performance of those feature vectors with different length of training set including 10, 15, 20 and 25 training set. Similar to last experiment, we randomly select the training set and repeat for 10 times for each test. The corresponding results are shown in Fig. 13. As can be seen, three features output similar classification results which all above 80%. Amongst different features, SVD outperforms others while PCA gives slightly worse result. This is because of SVD does not need to compute the covariance matrix that more fits our datasets (2D matrix for Doppler spectrogram). The physical features have better performance at low training number but with less improvement with higher training number.

**FIGURE 14.**
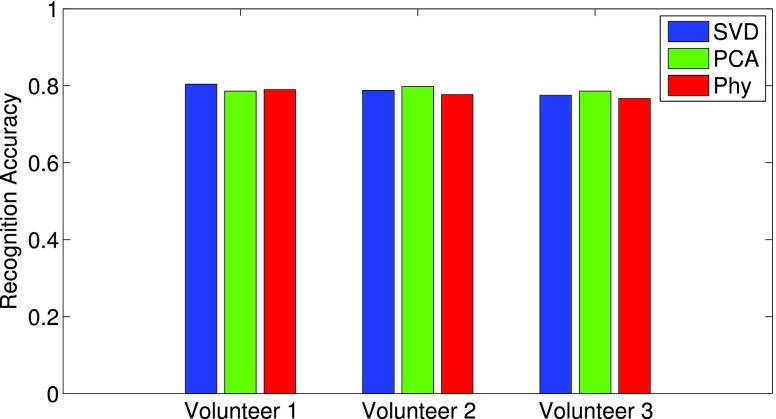
Classification accuracy for each volunteer by training from another volunteer.

Afterwards, we carry out an experiment for inter-subject comparison by using the testing sets from one volunteer and training sets from other volunteers. This procedure is more realistic as it classifies the activities for unknown people base on known people. In this experiment, we carried out tests for all volunteers (each uses 20 training sets from another two volunteers). The classification results are shown in Fig. 14 with three feature methods. As it can be seen, the classification performance is similar for three volunteers which are all around 80%. Understandably, those classification performances are lower than that in Fig. 13 (20 training set), due to the differences in Doppler pattern between training and testing volunteers. But still, those results indicate the proposed passive radar system has the ability of recognize activities from a new user base on the training from known datasets. This can be very helpful for e-Health applications.

## VI. Conclusions

Using passive radar as a non-contact sensor for breathing detection and activity recognition is a promising choice for healthcare applications. Because it does not require wearable sensors or special signal sources, offers unique benefits in long-term monitoring. This work proposed an adaptive passive Doppler radar based system which can capture the human breathing rate and recognize human activities. Also, a motion indicator has been implemented to automatically switch between those functions by solving the pulse detection problems. Experiments are performed in a controlled lab environment and present encouraging results. We have shown its consistency in capturing the Doppler change caused by inhalation and exhalation motions. Three scenarios, in terms of distance, orientation and separated signal source, have shown the system performs properly under various scenarios for breathing detection. The system has also been tested for activity recognition. Three feature vectors include SVD, PCA and physical features are used and tested with SVM classifier. The classification performance for those methods has been presented and shown with 85% achievable accuracy when 20 training sets are used. In addition, we perform the inter-subject classification by testing one person with datasets from other people. The results show about 80% accuracy (Fig. 14) that indicates the proposed concept is capable for real-world applications.

The presented experimental results show promising potential of using passive Doppler sensing technology in healthcare applications. Regarding the practical application in residential environments or clinical scenarios, two important aspects could be investigated as future work. **i) Multiple user cases:** The demonstrated system was developed for a single user situation. To make it more realistic, development of signal separation techniques and multiple user behavior models is needed. **ii) Sequential Models:** As human behaviors often present strong time correlation rather than time independence. Thus, introducing time sequential model such Hidden Markov Model (HMM) should help to improve activity recognition accuracy.
